# Cinnamic Acid and Its Derivatives Inhibit Fructose-Mediated Protein Glycation

**DOI:** 10.3390/ijms13021778

**Published:** 2012-02-08

**Authors:** Sirichai Adisakwattana, Weerachat Sompong, Aramsri Meeprom, Sathaporn Ngamukote, Sirintorn Yibchok-anun

**Affiliations:** 1 The Medical Food Research and Development Center, Department of Nutrition and Dietetics**,** Faculty of Allied Health Sciences, Chulalongkorn University, Bangkok 10330, Thailand; 2 Research Group of Herbal Medicine for Prevention and Therapeutic of Metabolic diseases, Chulalongkorn University, Bangkok 10330, Thailand; E-Mails: Kradat_pup@hotmail.com (W.S); Jamaea_p@hotmail.com (A.M.); ampam10@hotmail.com (S.N.); Siritorn.y@chula.ac.th (S.Y.); 3 Program in Clinical Biochemistry and Molecular Medicine, Department of Clinical Chemistry, Faculty of Allied Health Sciences, Chulalongkorn University, Bangkok 10330, Thailand; 4 Department of Pharmacology, Faculty of Veterinary Science, Chulalongkorn University, Bangkok 10330, Thailand

**Keywords:** cinnamic acid, glycation, advanced glycation end products, diabetic complications, fructose

## Abstract

Cinnamic acid and its derivatives have shown a variety of pharmacologic properties. However, little is known about the antiglycation properties of cinnamic acid and its derivatives. The present study sought to characterize the protein glycation inhibitory activity of cinnamic acid and its derivatives in a bovine serum albumin (BSA)/fructose system. The results demonstrated that cinnamic acid and its derivatives significantly inhibited the formation of advanced glycation end products (AGEs) by approximately 11.96–63.36% at a concentration of 1 mM. The strongest inhibitory activity against the formation of AGEs was shown by cinnamic acid. Furthermore, cinnamic acid and its derivatives reduced the level of fructosamine, the formation of N^ɛ^-(carboxymethyl) lysine (CML), and the level of amyloid cross β-structure. Cinnamic acid and its derivatives also prevented oxidative protein damages, including effects on protein carbonyl formation and thiol oxidation of BSA. Our findings may lead to the possibility of using cinnamic acid and its derivatives for preventing AGE-mediated diabetic complications.

## 1. Introduction

Chronic hyperglycemia causes non-enzymatic protein glycation by reducing sugars, such as glucose and fructose, which react with the free amino groups of protein to initiate a complex cascade of repeated condensations, rearrangements, oxidative modifications, ultimately forming advanced glycation end products (AGEs) [1,2]. It has been clearly demonstrated that the accumulation of AGEs in body tissue is the leading cause of several age-related degeneration, atherosclerosis and diabetic complications such as retinopathy, nephropathy and neuropathy [3–5]. A comprehensive review of the current scientific literature reveals that the inhibition of AGE formation is one of the therapeutic approaches to prevent the progression of diabetic complications [6]. For example, aminoguanidine (AG), a small synthetic hydrazine-like compound, has shown promising results in terms of inhibition of AGE formation, and has received the most interest from a Phase III clinical trials perspective. However, the trial was terminated because it has shown some toxicity problems in diabetic nephropathy, such as flu-like symptoms, gastrointestinal problems and anemia [7,8]. In this regard, the effort has been directed in finding effective phytochemical compounds from dietary plants, fruits and herbal medicines against protein glycation [9–12].

**Figure 1:**
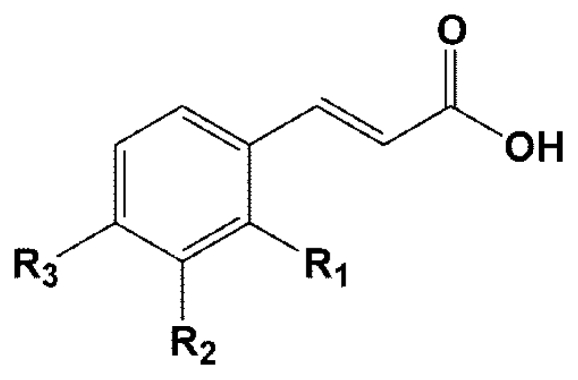

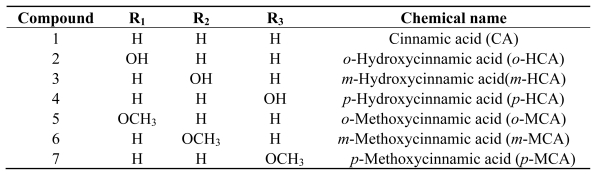
Structure of cinnamic acid and its derivatives.

Cinnamic acid and its derivatives are often characteristic of a plant species or even of a particular organ or tissue of that plant (Figure 1). Previous studies have shown the pharmacological properties of cinnamic acid and its derivatives, including hepatoprotective [13], anti-oxidant [14], and anti-diabetic activities [15]. To the best of our knowledge, studies regarding the protein glycation inhibitory effect of cinnamic acid and its derivatives have not been investigated. Therefore, the purpose of the present study was carried out to determine the inhibitory effect of cinnamic acid and its derivatives (Figure 1) against bovine serum albumin (BSA) in fructose-mediated non-enzymatic glycation. Moreover, we also examined the inhibitory effect of cinnamic acid and its derivatives on oxidation-dependent damages to BSA induced by fructose. Finally, we investigated their ability to inhibit the formation of the major chemical AG structure, N^ɛ^-(carboxymethyl)lysine (CML).

## 2. Results and Discussion

### 2.1. The Effect of Cinnamic Acid and Its Derivatives on AGEs Formation

**Figure 2:**
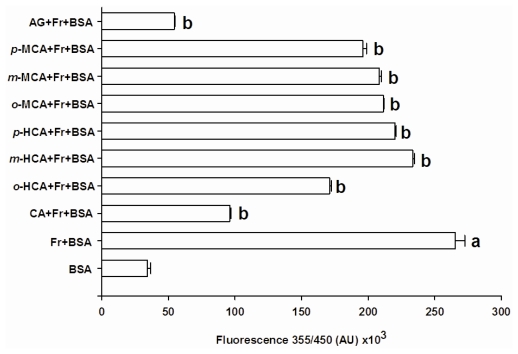
The effects of cinnamic acid and its derivatives (1 mM) and aminoguanidine (AG, 1 mM) on fluorescent AGEs formation in the BSA/fructose system. Each value represents the mean ± SEM (n=3). *^a^* *P < 0.05* compared to BSA, *^b^* *P < 0.05* compared to BSA +Fr (Fructose).

Figure 2 shows the effects of seven compounds at concentration of 1 mM on the total AGEs formation during 28 days of incubation. The fluorescent intensity of BSA incubated with fructose significantly increased about 7.8-fold when compared to BSA, indicating progressive formation of AGEs. When the glycation occurred in the presence of cinnamic acid, we observed that cinnamic acid significantly reduced the formation of AGEs by 63.36 ± 1.07%, as compared to BSA incubated with fructose. Upon the introduction of a hydroxyl group at the *ortho*-position of cinnamic acid (*o*-HCA), it was found that the percentage inhibition of AGE formation was 35.49 ± 1.73%. The addition of a hydroxyl residue in the cinnamic acid at the *meta*- or *para-* positions resulted in a significant loss of inhibitory activity (*m*-HCA = 11.96 ± 2.02%, and *p*-HCA = 16.95 ± 1.95%). Replacement of a hydroxyl residue in cinnamic acid by a methoxyl residue at the *ortho-* (*o*-MCA), *meta*- (*m*-MCA) or *para-*positions (*p*-MCA), produced respective percentage inhibitions of AGE formation of 20.25 ± 2.17%, 21.45 ± 1.67% and 26.10 ± 1.58%. However, these compounds were less potent than AG (79.46 ± 0.38%), which was used as control.

Protein glycation involves a series of complex reactions that occur between monosaccharides (glucose and fructose) and amino acids or proteins, which produce an unstable Schiff base, and then form an Amadori product such a fructosamine [1,2]. During the propagation reaction, the Amadori products react with the amino acids to form irreversible AGEs. According to the data obtained from this study, cinnamic acid was the strongest anti-glycating inhibitor among the compounds tested. Our findings also revealed that the presence of hydroxy or methoxy groups in cinnamic acid caused a significant decrease in the protein glycation inhibitory activity. The data presented here suggest that the presence of hydroxy or methoxy residues in cinnamic acid is not important for antiglycation activity. However, these findings are in contrast to our previous study reporting the structure-activity relationship of cinnamic acid and its derivatives on the inhibition of intestinal α-glucosidase [16]. It is worth noting that cinnamic acid was found to be a weak inhibitor against intestinal maltase and sucrase [16]. In addition, the introduction of a hydroxyl group at various positions of the cinnamic acid molecule can increase intestinal maltase and sucrase inhibitory activities, whereas the presence of methoxy residue on cinnamic acid demonstrates less potent activity than the presence of hydroxy residue. In addition, it has been reported that cinnamic acid has no insulin secreting activity, whereas the introduction of hydroxy residues to cinnamic acid causes an increase in insulin secreting activity from pancreatic β-cell [17]. Meanwhile, substitution of the hydroxy in cinnamic acid by the methoxy residue markedly increases the activity of insulin secretion. These findings suggest that adding hydroxy and methoxy residues in cinnamic acid is an important factor to exhibit insulinotropic activity.

### 2.2. The Effect of Cinnamic Acid and its Derivatives on the Level of Fructosamine and the Formation of CML

**Figure 3:**
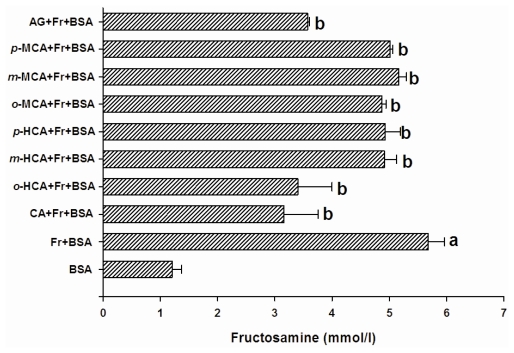
The effects of cinnamic acid and its derivatives (1 mM) and AG (1 mM) on the level of fructosamine in the BSA/fructose system. Each value represents the mean ± SEM (n = 3). *^a^* *P < 0.05* compared to BSA, *^b^* *P < 0.05* compared to BSA +Fr (Fructose).

**Figure 4:**
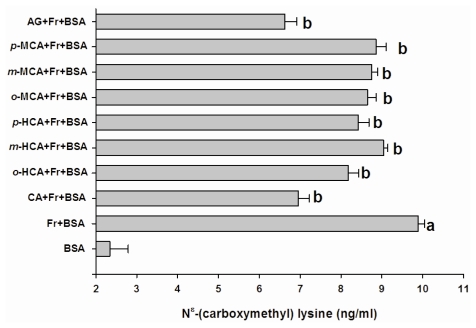
The effects of cinnamic acid and its derivatives (1 mM) and AG (1 mM) on the level of N^ɛ^-(carboxymethyl) lysine (CML) in the BSA/fructose system. Each value represents the mean ± SEM (n = 3). *^a^* *P < 0.05* compared to BSA, *^b^* *P < 0.05* compared to BSA +Fr (Fructose).

After day 28 of the experiment, the level of fructosamine in BSA incubated with fructose produced a 4.5-fold increase compared to BSA (Figure 3). The results showed that cinnamic acid significantly decreased the level of fructosamine by about 44.3%. In addition, 6 other compounds suppressed the elevation of fructosamine by approximately 5.5%–40.0%, whereas AG caused a decrease in the level of fructosamine by 35.8%. Figure 4 shows the effect of cinnamic acid and its derivatives on protein-bound CML formation. The results showed that the formation of CML in BSA incubated with fructose was significantly 4.26-times higher than BSA incubated without fructose. The addition of cinnamic acid and its derivatives to the solution could reduce CML-derived AGE by approximately 8.6–30.2%, whereas AG inhibited the formation of CML by 67.9% when compared to BSA incubated with fructose.

The determination of BSA fructosamine levels is used to monitor the accumulation of early (Amadori) glycation products. The results demonstrated that the reduced level of fructosamine by cinnamic acid and its derivatives was associated with the decreased formation of AGEs. Our results suggest that cinnamic acid and its derivatives have an inhibitory effect on Amadori production, resulting in the prevention of conversion into AGEs. N^ɛ^-(carboxymethyl) lysine (CML) is the most used marker for AGEs in humans [18]. This product can accumulate in tissues, causing increased inflammation, reduced antioxidant defense, and accelerated micro- and macrovasculopathies [19]. The findings indicate that cinnamic acid and its derivatives are effective in reducing CML formation associated with reduced levels of fructosamine.

Cinnamon, used variously in food as a herb or spice, has been shown to ameliorate the symptoms of metabolic syndromes, such as insulin resistance, elevated levels of glucose and lipids and decreased antioxidant [20]. There are some reports in the literature showing that cinnamon extract exhibits potent inhibition of protein glycation [9,10]. The constituents of cinnamon bark are mainly cinnamic acid, cinnamaldehyde and cinnamic alcohol [21]. Therefore, it is possible that the reduced protein glycation of cinnamon extract observed in the previous study can be best explained by the inhibitory activity of cinnamic acid.

### 2.3. The Effect of Cinnamic Acid and Its Derivatives on the Level of Amyloid Cross-β Structure

**Figure 5:**
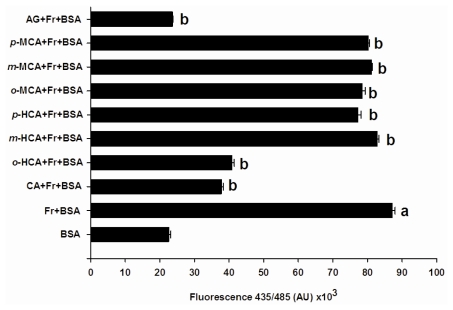
The effects of cinnamic acid and its derivatives (1mM) and AG (1 mM) on thioflavin T fluorescence in the BSA/fructose system. Each value represents the mean ± SEM (n = 3). *^a^* *P < 0.05* compared to BSA, *^b^* *P < 0.05* compared to BSA +Fr (Fructose).

As shown in Figure 5, the level of amyloid cross-β structure in BSA was determined by using thioflavin T, a fluorescent dye that specifically binds with fibrous structures. The fluorescent intensity of BSA incubated with fructose resulted in a 3.86-fold increased response, as compared with BSA incubated without fructose, suggesting that the protein glycation progressively induced the formation of amyloid cross-β structure in BSA. The results showed that cinnamic acid markedly decreased the fluorescent intensity of BSA incubated with fructose. Furthermore, reduced fluorescent intensity was also observed in BSA by incubation of fructose and the series of hydroxycinnamic acids (*o*-HCA, *m*-HCA, and *p*-HCA), whereas the presence of the series of methoxycinnamic acids (*o*-MCA, *m*-MCA, and *p*-MCA could decrease fluorescence intensity slightly.

Glycation is a key mechanism to induce the conformational changes of protein by increasing the level of amyloid cross β-structure, which plays a fundamental role in the protein aggregation. Studies reveal that the deposition of protein aggregation has been associated with the progression of several debilitating degenerative diseases including hemodialysis amyloidosis, diabetes, Parkinson’s disease and Alzheimer’s disease [22–24]. Notably, accumulation of protein aggregation causes pancreatic islet amyloidosis, which directly induces β-cell damage and impaired insulin secretion [25]. Our data clearly establish that cinnamic acid and its derivatives suppress in the level of amyloid cross β-structure of BSA. This beneficial effect of cinnamic acid and its derivatives may help to reduce a risk of developing debilitating degenerative diseases in diabetic patients.

### 2.4. The Effect of Cinnamic Acid and its Derivatives on Glycation-Induced Protein Oxidation

Table 1 shows the results for available free thiol groups and oxidative modification of BSA on cinnamic acid and its derivatives. A significant decrease in free thiol groups was observed in BSA incubated with fructose, indicating that protein glycation modified thiol groups to form disulfide in BSA. It found that cinnamic acid and its derivatives significantly reduced the oxidation of thiol groups by approximately 9.5–28.6%, whereas AG also protected the loss of protein thiol groups about 22.2%, as compared to BSA incubated with fructose.

The addition of fructose to the BSA solution for 28 days significantly increased the extent of protein carbonyl formation, compared to BSA in the absence of fructose. It found that cinnamic acid and its derivatives suppressed protein carbonyl formation by approximately 18.2–25.7% when compared to BSA incubated with fructose. In addition, AG reduced protein carbonyl formation in BSA incubated with fructose by 30.9%.

In general, glycation and AGE-induced toxicity are known to be associated with increased free radical production [2]. In particular, the process of oxidative degradation of Amadori intermediates can generate free radicals, causing damage by oxidizing proteins [26]. Major molecular modifications of protein structural changes can be investigated by protein carbonyl formations and the loss of protein thiol groups [27]. Moreover, the investigation of thiol group in BSA is the direct reflection of excess free radical generation [27]. The marked increase in protein carbonyl formation and the oxidation of thiols in BSA were observed fructose incubation. Our findings show that the addition of cinnamic acid and its derivatives together with fructose to a BSA solution significantly suppresses the protein carbonyl formation and oxidation of thiols. There is considerable evidence to support the argument that trapping free radicals and reactive carbonyl group formation by antioxidant compounds is a strategy for the inhibition of protein glycation [6]. Recently, cinnamic acid and its derivatives have shown favorable effects in antioxidant properties [10]. It can be assumed that the mechanism of cinnamic acid and its derivatives for lowering protein glycation may be related to their antioxidant activity. However, the antioxidant activity of cinnamic acid and its derivatives may not be the only reason to explain the antiglycating mechanism. Biochemical mechanisms of anti-glycation reactions have been recently proposed, such as breaking the cross-linking structures in AGEs that have been formed, blocking the carbonyl or dicarbonyl groups in reducing sugars, Schiff bases or Amadori and inhibiting the formation of late-stage Amadori products [6]. Further comprehensive studies of cinnamic acid and its derivatives are required to evaluate the antiglycating mechanisms described above.

## 3. Experimental Section

### 3.1. Chemicals

*o-*Hydroxycinnamic acid (*o*-HCA), *m-*hydroxycinnamic acid (*m*-HCA) and *p-*hydroxycinnamic acid (*p*-HCA) were purchased from Fluka (St. Louis, MO). *o-*Methoxycinnamic acid (*o*-MCA), *m-*methoxycinnamic acid (*m*-MCA) and *p-*methoxycinnamic acid (*p*-MCA) were purchased from ACROS (Pittsburgh, PA, USA). Cinnamic acid, bovine serum albumin (BSA, fraction V), aminoguanidine hydrochloride, guanidine hydrochloride, thioflavin T, 5,5′-Dithiobis(2-nitrobenzoic acid), nitroblue-tetrazolium and L-cysteine were purchased from Sigma-Aldrich Co. (St. Louis, MO, USA). Fructose and 2,4-dinitrophenyl hydrazine were purchased from Ajax Finechem (Taren Point, Australia). Trichloroacetic acid was purchased from Merck (Darmstadt, FR, Germany). OxiSelect™ N^ɛ^-(carboxymethyl) lysine (CML) ELISA kit was obtained from Cell Biolabs (San Diego, CA, USA). All other chemicals and solvents used in this study were of analytical grade.

### 3.2. Glycation of Bovine Serum Albumin (BSA)

The glycated BSA formation was undertaken in accordance with a previous method with a minor modification [28]. In brief, BSA (10 mg/ml) was incubated with 500 mM fructose in a 0.1 M phosphate buffered-saline, pH = 7.4 containing 0.02% sodium azide in the dark at 37 °C for 28 days. Before incubation, cinnamic acid and its derivatives and aminoguanidine (final concentration: 1 mM) were added to the mixtures. Dimethylsulfoxide (DMSO) was used for a solvent for this study (final concentration: 4.0%). The glycated BSA formation was measured by using fluorescent intensity at an excitation wavelength 355 nm and emission wavelength 460 nm.

### 3.3. Determination of Fructosamine

The concentration of fructosamine, the Amadori product, was measured by using the nitroblue-tetrazolium (NBT) assay [29]. Briefly, glycated BSA was incubated with 0.5 mM NBT in 0.1 M carbonate buffer, pH = 10.4 at 37 °C. The absorbance was measured at 530 nm. The concentration of fructosamine was calculated using the different absorption at the time point of 10 and 15 min, and compared to 1-deoxy-1-morpholino-fructose (1-DMF) as the standard.

### 3.4. Determination of N^ɛ^-(Carboxymethyl) Lysine (CML)

N^ɛ^-(carboxymethyl) lysine (CML), a major AGE structure, was determined by using enzyme linked immunosorbant assay (ELISA) kit. The concentration of CML was calculated by using the standard CML-BSA curve from the assay kit.

### 3.5. Determination of Amyloid Cross β Structure

Thioflavin T, a marker of amyloid cross β structure, was measured according a previous method with minor modifications [30]. Briefly, glycated BSA was incubated with 32 μM thioflavin T in 0.1 M PBS, pH = 7.4. After 60 min incubation, the fluorescence intensity was measured at an excitation wavelength of 435 nm and an emission wavelength of 485 nm.

### 3.6. Determination of Protein Carbonyl Content

Carbonyl group in glycated BSA, a marker for protein oxidative damage, was assayed according to a previous method [31]. In brief, glycated BSA was incubated with 10 mM 2,4-dinitrophenylhydrazine (DNPH) in 2.5 M HCl at room temperature for 60 min. Afterwards, glycated BSA was precipitated by 20% (w/v) trichloroacetic acid (TCA), left on ice for 5 min, and centrifuged at 10,000g for 10 min at 4° C. The pellet was washed three times using 500 μL of 1:1 (v/v) ethanol:ethyl acetate mixture. The final pellet was dissolved in 6 M guanidine hydrochloride. The absorbance was read at 370 nm. The protein carbonyl group concentration was calculated by using absorption coefficient (ɛ = 22,000 M^−1^·cm^−1^). The results were expressed as nmol carbonyls/mg protein.

### 3.7. Thiol Group Estimation

The free thiol groups of glycated BSA were measured according to Ellman’s assay with slight modifications [32]. Briefly, glycated BSA was incubated with 5 mM 5,5′-dithiobis(2-nitrobenzoic acid) (DTNB) in 0.1 M PBS, pH 7.4 for 15 min. Thereafter, the absorbance was measured at 412 nm. The free thiol concentration was calculated by using L-cysteine as standard curve. The results were expressed as nmol/mg protein.

### 3.8. Statistical Analysis

The results were expressed as the mean ± standard error of the mean (SEM) (*n* = 3). The statistical significance of the results was evaluated by using one-way ANOVA. The least significant difference (LSD) test was used for mean comparisons, and *P* < 0.05 was considered to be statistically significant.

## 4. Conclusions

We found that cinnamic acid and its derivatives could effectively protect BSA from fructose-mediated protein glycation *in vitro*. They also reduced the level of fructosamine, the formation of CML and the amyloid cross β-structure in BSA. In addition, cinnamic acid and its derivatives significantly decreased the protein carbonyl content and increased the level of protein thiol. These findings may be applied for prevention or management of AGE-mediated pathologies, particularly for those who are at risk of developing diabetic complications.

## Figures and Tables

**Table 1:** The effects of cinnamic acid and its derivatives (1 mM) and AG (1 mM) on thiol group content and protein carbonyl formation of fructose-modified BSA.

Compound	Thiol group (nmol/mg protein)	Protein carbonyl content (nmol/mg protein)
BSA	0.89 ± 0.01	0.25 ± 0.02
BSA+Fr	0.63 ± 0.03 a	2.30 ± 0.08 a
BSA+Fr+ CA	0.75 ± 0.06 b	1.90 ± 0.07 b
BSA+Fr+ *o*-HCA	0.71 ± 0.01 b	1.88 ± 0.07 b
BSA+Fr+ *m*-HCA	0.69 ± 0.04 b	1.97 ± 0.05 b
BSA+Fr+ *p*-HCA	0.80 ± 0.02 b	1.84 ± 0.05 b
BSA+Fr+ *o*-MCA	0.76 ± 0.02 b	1.75 ± 0.07 b
BSA+Fr+ *m*-MCA	0.76 ± 0.03 b	1.84 ± 0.04 b
BSA+Fr+ *p*-MCA	0.74 ± 0.01 b	1.71 ± 0.10 b
BSA+Fr+AG	0.81 ± 0.02 b	1.59 ± 0.77 b

Each value represents the mean ± SE (*n* = 3).

a *P <* 0.05 compared to BSA,

b *P <* 0.05 compared to BSA + Fr (Fructose).
